# Work-related musculoskeletal disorders and risk factors among weavers: A cross-sectional study

**DOI:** 10.4102/sajp.v79i1.1904

**Published:** 2023-07-31

**Authors:** Pegdwendé A. Kaboré, Bénédicte Schepens

**Affiliations:** 1 Laboratory of Physiology and Biomechanics of Locomotion-Institute of Neuroscience, Faculty of Movement and Rehabilitation Sciences, Catholic University of Louvain, Louvain-la-Neuve, Belgium; 2 National Center for Orthopedic Equipment of Burkina, Ouagadougou, Burkina Faso

**Keywords:** key indicator method, ergonomic, Nordic questionnaire, workplace, handloom weavers, informal sector

## Abstract

**Background:**

Work-related musculoskeletal disorders (WRMSDs) are a major health issue for low-income countries because of their high prevalence among workers and on account of the scarcity of ergonomic preventative measures in the workplace.

**Objectives:**

To determine the prevalence of WRMSDs among weavers, assess their consequences, and analyse the associated occupational risk factors.

**Method:**

A cross-sectional survey was carried out among 257 handloom weavers using the Nordic questionnaireand the working conditions were assessed through a descriptive analysis using the key indicator method.

**Results:**

The WRMSDs annual prevalence was 85% in all parts of the body, 71% for the low back region, 41% for the shoulders, and 37% for the knees. One quarter of the weavers indicated having stopped work for 1–30 days because of their low back pain (LBP). The prevalence of WRMSDs was associated with the number of hours worked per day, the years of experience, and age. Long working hours, load carrying (> 20 kg – 25 kg), awkward postures, repetitive limb movements, and unfavourable environmental conditions were identified as occupational risk factors.

**Conclusion:**

Work-related musculoskeletal disorders are common among weavers and LBP is the most frequently cited disorder and the primary reason for work interruptions and a decrease of activities. The prevalence of WRMSDs is associated with professional and personal factors. Actions based on ergonomic rules are necessary to prevent WRMSDs.

**Clinical implications:**

Our study highlights the issue of WRMSDs and the need for prevention in the informal sector, which constitutes the major part of economic activity in low-income countries.

## Introduction

Work-related musculoskeletal disorders (WRMSDs) are:

[*D*]isorders of the musculoskeletal system for which work activity may play a role in the genesis, maintenance, or aggravation. These disorders can lead to functional discomfort or pain, or even a limitation of activity. (INRS 2020)

They may be associated with daily working hours, work experience, workstation postures or personal factors such as age, gender, and medical history (Koiri 2020; Muhamad Ramdan, Candra & Rahma Fitri 2020; Nag, Vyas & Nag 2010; SPF-EMPLOI 2013). A report by the International Labour Organisation (ILO) ranked WRMSDs as the leading cause of absenteeism in the workplace among work-related illnesses with over 160 million people affected per year (ILO 2013). The prevalence of WRMSDs varies among countries and sectors; workers of the informal sector appear to be more exposed than those of the formal sector (Durlov et al. 2014; Siddiqui et al. 2021). In low-income countries, musculoskeletal disorders are a major health issue, because of their high prevalence among workers and on account of the scarcity or absence of ergonomic prevention measures in the workplace (Durlov et al. 2014).

Weaving is a widespread occupation in several low-income countries, such as India, Bangladesh, Indonesia, and Burkina Faso (BRMN 2018; Muhamad Ramdan et al. 2020; Rahman et al. 2017; Siddiqui et al. 2021). Burkina Faso is a leading cotton producer in Africa and this sector is one of the country’s primary resources (FAO/EST 2022). Hand looming is an independent activity, part of the informal sector, and more than 20 500 Burkinabe women were involved in cotton processing through weaving in 2004 (BRMN 2018).

Weaving activity is divided into three phases: preparation of the yarn or warping, dyeing, and weaving. The preparation of the yarn can be done either by walking and unwinding the yarn around pegs planted in the ground or by staying on the spot and unwinding the yarn around a wooden spinning top. For dyeing, the threads are first soaked in basins containing hot water mixed with toxic chemical dyes. Thereafter, the threads are removed from the mixture, rinsed, and spread out to dry. Weaving is the longest lasting activity; it is carried out in a seated position with alternating and rhythmic movements of the limbs: the lower limbs operate the two pedals of the loom and the upper limbs pass the shuttle through a space created by the heddles as a result of the action of the pedals.

In Asia, weaving is considered as one of the most strenuous and demanding occupations and a high-risk for the development of musculoskeletal disorders. Artisan weavers demonstrate a high prevalence of WRMSDs in all parts of the body and the lower back is among the areas most affected (Durlov et al. 2014; Muhamad Ramdan et al. 2020; Rahman et al. 2017).

In Burkina Faso, the consequences of weaving on the musculoskeletal health of weavers are still poorly understood because of the lack of research on the issue and that weaving is undertaken in the informal work sector. A recent study carried out among weavers in Burkina Faso showed a high prevalence of WRMSDs; however, the issues of the consequences of WRMSDs and the physical and environmental risk factors involved were not investigated (Sawadogo et al. 2020).

Firstly, our study aimed to assess the prevalence of WRMSDs among weavers and their consequences on work, and secondly, to highlight physical risk factors and environmental constraints.

## Methods

Our descriptive cross-sectional study was conducted from June 2020 to October 2020, following the STROBE (STrengthening the Reporting of OBservational studies in Epidemiology) guidelines (Von Elm et al. 2008). Purposive sampling was used to select the participants based on geographical distribution.

**FIGURE 1:**
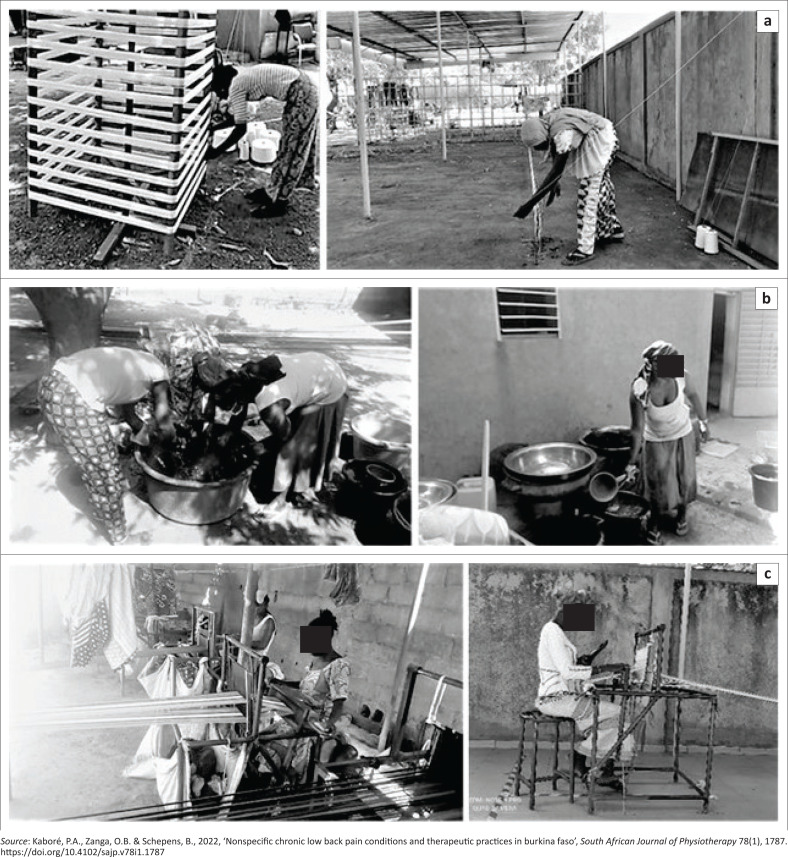
An illustration of weaving activity in the workplace in Ouagadougou city. (a) Preparation of the yarn by spinning top (right) and by walking (left); (b) dyeing; (c) weaving.

Our study was carried out among 247 handloom weavers at their workplace in the Ouagadougou area (Burkina Faso) (Figure 1). Participants were female, using horizontal pedal looms, and having at least 1 year of practice in the profession.

### Measurement and data collection

Weavers were firstly interviewed about their musculoskeletal symptoms and secondly, the physical occupational risk factors of the weaving activity were identified.

The purpose of our study was explained to each participant and informed consent was obtained in the presence of a witness according to standard ethical rules.

Weavers were interviewed using the French version of the Nordic Questionnaire (NQ) to assess the prevalence of WRMSDs (Kuorinka et al. 1987). The NQ is an epidemiological questionnaire on the musculoskeletal health of workers where prevalence is estimated over the life course, over 1 year and over the last 7 days by anatomical zone (neck, shoulders, elbows, wrists and hands, upper back, lower back, hips and thighs, knee, ankles, and feet). The NQ is divided into three sections. Section 1 (17 items): provides general information about the worker and his or her occupation, such as the age, height, weight, working experience, work absences, and decrease in annual activities related to lower back pain. Section 2 (27 items): provides questions on the prevalence and work stoppages over the last 12 months for each part of the body, and Section 3 (81 items): provides information on WRMSDs by body part. The sensitivity and the specificity of the questionnaire are 83.5% and 81.1%, respectively (Descatha et al. 2007).

The questionnaire was initially translated into the local language ‘Mooré’ using the forward and backward translation method (Beaton et al. 2000). The translation was carried out separately by a teacher of the Mooré language and a journalist in the local Mooré language, and then those translations were pooled together with the one of the authors of our study. A back-translation into French of this version was carried out by another translator totally blind to the initial version. Differences in translation concerned the tense used in the formulation of one item in Section 3 of the questionnaire and was resolved by consensus. The questionnaire was then tested on 10 non-weaver and 10 weaver women to ensure that the questions were well understood and to evaluate the duration of the interview (10 min – 15 min). The analysis of this last step was performed by the first author and the translators and no discrepancies were found among the respondents to the questionnaire.

The physical occupational risk factors were identified using the Key Indicator Method (KIM) for the three sub-activities: preparation of the yarn or warping, dyeing, and weaving. The KIM was developed by the German Federal Institute for Occupational Safety and Health to document the main physical workload indicators with ease and to assess the probability of physical overload (BAUA 2019). Possible consequences for health as well as the resulting need for action are identified (BAUA 2019; Klussmann et al. 2017). The method identifies the physical occupational risk factors considering the duration of the activity and specific key indicators such as body postures, frequency of movements and lifting, effective weight of loads carried, body movement, force transfers, unfavourable working conditions, and work organisation. The purpose of using the KIM was to facilitate a detailed descriptive analysis of the risk factors identified in the weaving profession by activity (warping, dyeing and weaving).

Several screening tools are available depending on the physical workload. The preparation of the yarn by walking and by spinning was analysed using the KIM for assessing and designing physical workloads with respect to Body Movement (KIM-BM) and the KIM for assessing and designing physical workloads with respect to Awkward Body Postures (KIM-ABP). The dyeing activity was analysed using the KIM for assessing and designing physical workloads with respect to manual Lifting, Holding and Carrying of loads ≥ 3 kg (KIM-LHC). The weaving activity was analysed using the KIM-ABP and the KIM for assessing and designing physical workloads during Manual Handling Operations (KIM-MHO).

The prevalence of musculoskeletal symptoms, the cessation of work, the reduction of annual activities related to low back pain (LBP), and the physical occupational risk factors were the primary variables. Socio-anthropometric parameters such as age, height, weight, working experience, work absences, and decrease in annual activities related to LBP were secondary variables.

### Statistical analysis

The results were analysed using Statistical Package for Social Sciences (SPSS) 27.0. Descriptive statistics and frequency tables were utilised for demographic and occupational characteristics. The quantitative variables, age, body mass index (BMI), work experience, hours of work per day were normally distributed (Kolmogorov–Smirnov normality test). Fisher’s exact test was used to analyse the dependence between WRMSDs and the number of working hours per day, the number of years of work experience, and the age. The odds ratios between the prevalence of WRMSDs and working hours per day, work experience and age were calculated in reference to the central tendencies of the sample. The level of significance was set at 0.05.

## Results

### Characteristics of the sample

The average age of the weavers was 39 ± 9 years (Table 1). Half of the weavers had at least a height of 1.62 m and a BMI less than 24.2. Half of the weavers had more than 10 years of experience. The average working time per day was 8 ± 2 h, 50% of the sample worked more than 54 h per week and 75% worked more than 42 h per week. The number of working days per week was on average 6 ± 1, and 25% of the sample worked 7 days a week.

**TABLE 1:** Characteristics of the sample (*n* = 247).

Variable	Min-Max	Mean ± s.d.	Percentile		
			25th	50th	75th
Age (year)	18–63	39 ± 9	34	38	45
Height (m)	1.44–1.85	1.62 ± 0.06	1.58	1.62	1.65
BMI (kg/m²)	16.0–38.0	25 ± 5	21.0	24.2	28.3
Work experience (year)	1–45	11 ± 8	6	10	14
Hours of work per day	3–12	8 ± 2	7	8	9
Hours of work per week	18–77	51 ± 12	42	54	60
Working days per week	5–7	6 ± 1	6	6	7

Note: Body mass index (BMI) is calculated as the weight (kg) divided by the square of the height (m^2^).

Min, minimum; Max, maximum; s.d., standard deviation; m, metre; kg, kilogram.

### Work-related musculoskeletal disorders prevalence and consequences

All area’s body prevalence of WRMSDs was 89.9% for the entire lifetime, 85.4% for the last 12 months, and 34.8% for the last 7 days (Table 2). The low back was the most frequently cited region with a prevalence of 74.1%, 70.9%, and 45.3%, respectively, for lifetime, annual and last 7 days. The annual prevalence for the shoulders and the knees were 40.5% and 36.8%, respectively. The wrists and hands (21.1%) and the elbows (10.1%) were the least mentioned areas.

**TABLE 2:** Prevalence and consequences of musculoskeletal disorders of weavers (*n* = 247).

Area of the body	Prevalence (%)			Number of weavers (%)			
	Entire life	Last 12 months	Last 7 days	Reduction of activities†	Cessation of activities*		Consultation with health professional†
					1–30 days	> 30 days	
Neck	27.4	23.5	10.5	7.7	8.5	0.0	0.8
Shoulders	40.9	40.5	18.2	12.6	7.7	0.8	5.3
Elbows	11.2	10.1	5.2	0.8	0.4	0.0	0.0
Wrists and hands	23.1	21.1	12.1	4.5	4.4	1.2	2.4
Upper back	26.7	25.1	15.4	7.7	8.1	1.2	6.1
Low back	74.1	70.9	45.3	27.1	24.3	1.2	13.0
Hips and thighs	27.1	27.1	15.4	6.9	5.3	0.8	2.4
Knees	42.9	36.8	18.6	8.1	3.2	0.0	5.3
Ankles and feet	26.3	25.5	17.8	4.9	3.2	0.0	3.2
All areas of body	89.9	85.4	65.2	n/a	n/a	n/a	n/a

n/a, not applicable.

† , during the last 12 months.

During the last 12 months, 13% of the weavers consulted a health professional because of their back pain. The lumbar spine complaints were the main reasons for a decrease and cessation of the weavers’ usual activities (work and home): 27.1% of the weavers decreased their usual activities and 24.3% stopped work for between 1 and 30 days.

### Correlation analysis between the prevalence of work-related musculoskeletal disorders and the characteristics of the occupation

The prevalence of upper back WRMSDs was associated with the number of working hours per day (*p* = 0.029): weavers who worked 8 h or more a day were twice as likely to develop upper back WRMSDs compared with those who worked less than 8 h (OR = 2.00, 95% CI: 1.11–3.61) (Table 3).

**TABLE 3:** Correlation analysis between the annual prevalence of work-related musculoskeletal disorders and the number of hours worked per day, work experience and age.

Variable	Neck		Shoulder		Elbow		Wrist hand		Upper back		Low back		Hip thigh		Knee		Ankle feet		All body	
	Odds ratio	95% CI	Odds ratio	95% CI	Odds ratio	95% CI	Odds ratio	95% CI	Odds ratio	95% CI	Odds ratio	95% CI	Odds ratio	95% CI	Odds ratio	95% CI	Odds ratio	95% CI	Odds ratio	95% CI
**Hours of work per day: 8 h and more per day versus less than 8 h**	1.45	0.79–2.66	1.55	0.90–2.64	1.39	0.60–3.24	0.69	0.35–1.36	2.00 *	1.11–3.61	1.19	0.66–2.14	0.98	0.54–1.78	1.34	0.78–2.31	1.76	0.98–3.18	1.86	1.41–2.80
**Work experience: 10 years or more versus less than 10 years**	1.19	0.66–2.14	1.20	0.72–2.00	1.03	0.44–2.37	2.09 *	1.12–3.89	2.22 **	1.24–3.99	1.94 **	1.54–2.63	1.18	0.67–2.07	0.68	0.40–1.15	0.75	0.42–1.35	1.42 *	0.68–2.96
**Age: 40 years and older versus less than 40 years**	1.38	0.97–2.12	1.44	1.06–2.10	1.29	0.83–2.41	0.71	0.37–1.37	0.69	0.38–1.28	1.74 *	1.20–2.71	1.36	0.98–2.05	1.89 *	1.32–2.87	1.31	0.95–1.99	1.70	1.07–3.03

Note: Values in bold represent the significant association.

CI, confidence interval.

* , *p* < 0.05;

** , *p* < 0.01.

The prevalence of WRMSDs, all areas of the body, was associated with work experience (*p* = 0.022): weavers who had worked 10 years or more were 1.42 times more likely to develop WRMSDs in the last 12 months compared with those who had worked less than 10 years (OR = 1.42, 95% CI: 0.68–2.96). There was also an association between the prevalence of low back WRMSDs and the number of years worked (*p* = 0.004): weavers who had worked 10 years or more were 1.94 times more likely to develop low back WRMSDs compared with those who had worked for less than 10 years (OR = 1.94, 95% CI: 1.54–2.63). The prevalence of wrists and hands and of upper back WRMSDs was associated with the working experience (number of years worked) (*p* = 0.027, *p* = 0.008, respectively) (Table 3).

The prevalence of low back WRMSDs was associated with age (*p* = 0.020): weavers aged 40 years and older were 1.74 times more likely to develop low back WRMSDs compared with those younger than 40 years (OR = 1.74, 95% CI: 1.20–2.71). There was also an association between the prevalence of knees WRMSDs and age (*p* = 0.030). Weavers aged 40 years and older were 1.89 times more likely to develop knees WRMSDs compared with those younger than 40 years (OR = 1.89, 95% CI: 1.32–2.87) (Table 3).

### Identification of variables of interest associated with work-related musculoskeletal disorders

Physical and environmental risk factors were identified for all phases of the weaving activity. For the preparation of yarn by walking, the key indicators identified were ‘body movement without using equipment’ and ‘unfavourable working conditions’. The corresponding risk factors were moderate walking (3 km/h – 5 km/h) without equipment, trunk frequently flexed or twisted, and permanent climatic influence such as heat and wind (Table 4). For the preparation of yarn by spinning, the key indicators identified were ‘loads on the back/body posture’, ‘load on shoulders and arms’, ‘load on knees and legs’, and ‘unfavourable working conditions’ (Table 4). The corresponding risk factors were a torso being flexed (> 60°) in a standing position, elevated arms, hands above shoulder level, constant standing, twisting and/or lateral tilting of the trunk. The postures and positions were maintained for more than one quarter of the time of the activity (Table 4).

**TABLE 4:** Key indicators identified for each activity and corresponding risk factors.

Key indicators	Risk factors
**Yarn preparation by walking (KIM-BM)**	
Body movement without using equipment	Walking at a moderate pace (3 km h^-1^ – 5 km h^-1^) without using equipment and carrying load
	Trunk clearly inclined forward and/or twisting, and/or lateral inclination of the trunk identifiable (frequently)
Unfavourable working conditions	Constant extreme climatic influences: heat, wind
**Yarn preparation by spinning (KIM-ABP)**	
Loads on the back/body posture	Torso being severely inclined forward (> 60°) in a standing position, up to half amount of time of activity (frequently)
Load on shoulders & arms	Arms raised, hands above shoulder level in a standing position, up to one quarter amount of time of activity
Load on knees and legs	Constant standing, also interrupted by walking a few steps, up to three quarters amount of time of activity
Unfavourable working conditions	Frequent twisting and/or lateral inclination of the trunk identifiable
**Dyeing (KIM-LHC)**	
Effective load weight	20 kg – 25 kg
Load handling conditions	Load handled temporarily with one hand, uneven load distribution between the two hands
Body posture	Frequent bending of the trunk, load or hands sometimes away from the body
Unfavourable working conditions	Difficulties because of holding/carrying: Load to be held 5 s – 10 s or carried over a distance 2 m – 5 m
**Weaving (KIM-MHO, KIM-ABP)**	
Type of force exertion finger/hand	Very low/low forces, average holding time 16 s/min – 30 s/min and average movement frequencies 16–30 times/min
Hand-arm position and movement	Frequent positions or movements of joints at the limit of the movement ranges
Unfavourable working conditions	Frequent impaired detail recognition because of dazzle or excessively small details, difficult conditions such as draught, moisture, noise
Body posture/movement	Predominantly sitting with occasional walking. Trunk with slight inclination towards the work area. Occasional twisting and/or lateral inclination of the trunk identifiable. Occasional deviations from good ‘neutral’ head posture/movement
Work organisation	No or hardly any variation of the physical workload situation because of other activities (including other types of physical workload)
Loads on the back /body posture	Sitting in forced postures, torso being moderately inclined forward, mostly looking permanently towards the work area more than three quarter of the time of activity
Load on shoulders and arms	Arms raised, hands below shoulder level or at a distance from the body in a sitting position without the arms being supported
Unfavourable working conditions	Constant narrow space for movement

KIM, key indicator method; KIM-BM, key indicator method-body movement; KIM-ABP, key indicator method-awkward body postures; KIM-LHC, key indicator method-lifting, holding and carrying of loads; KIM-MHO, key indicator method manual-handling operations.

For the dyeing activity, the key indicators identified were ‘effective load weight’, ‘load handling conditions’, ‘body posture’, and ‘unfavourable working conditions’. The corresponding risk factors were carrying a load between 20 kg and 25 kg, temporary handling of the load with one hand, uneven load distribution between the two hands, frequent flexion of the trunk with the load in the hands sometimes far from the body, the load to be held for 5 s – 10 s or to be carried over 2 m to 5 m (Table 4).

For the weaving activity, the KIM-MHO analysis identified the following key indicators: ‘type of force exertion in the finger/hand area’, ‘hand-arm position and movement’, ‘unfavourable working conditions’, ‘body posture/movement’, and ‘work organisation/temporal distribution’. The KIM-ABP identified the following key indicators: ‘loads on the back/body posture’, ‘load on shoulders and arms’ and ‘unfavourable working conditions’. The corresponding risk factors are presented in Table 4.

## Discussion

The objectives of our study were to assess the prevalence of WRMSDs and their consequences and to analyse in parallel the associated occupational risk factors.

Eighty-five percent of the weavers declared WRMSDs during the last 12 months, particularly in the low back region (71%). These results are comparable to the ones evidenced by a recent study carried out among Burkina Faso weavers showing an annual prevalence of 98% with a prevalence of 77% for the low back region (Sawadogo et al. 2020). Similar prevalences are also reported for carpet weavers in India 68% (Durlov et al. 2014), 79% (Pavana & Mica 2021), 67% (Naz, Kwatra & Ojha 2015), in Bangladesh 66% (Rahman et al. 2017) and in Iran 68% (Nazari et al. 2012). The prevalence of low back WRMSDs among weavers is higher than that observed in other occupations in Burkina Faso, such as nursing (57%) and physical education teachers (59%) (Nana et al. 2022; Ouédraogo et al. 2010).

Although there are differences among anatomical areas, all areas of the body are affected: the low back region firstly, the shoulder secondly, then the knee, hips and thighs and upper back (Table 2). There seems to be a consensus all around the world regarding these main affected regions (Durlov et al. 2014; Nazari et al. 2012; Sawadogo et al. 2020). This high prevalence of WRMSDs in all body areas in several countries and continents highlights that weaving is a demanding occupation exposing workers to health risk factors (Nag et al. 2010).

However, despite the high prevalence of WRMSDs, few weavers consult a health professional (Table 2). Burkina Faso is a low-income country where most workers of the informal sector are poor and cannot read or write. Indeed, the gross domestic product (GDP) per capita in Burkina Faso is less than 900 € (Euro) and 58% of women have no educational level (EDS 2021). A lack of information and/or difficulties in accessing healthcare system could explain this low attendance.

The sale of Faso Dan Fani is a source of income for the Burkinabé women, enabling them to look after their families and schooling of their children; however, more than a quarter of the weavers reduced their activities and/or interrupted their occupation because of their back pain in the year preceding the survey. As the weaving belongs mainly to the informal sector, it is difficult to precisely identify the economic consequences of work absence, but individual consequences are not negligible in terms of income and family status in a country where the status of women and poverty are challenging. Moreover, the consequences of LBP observed here could be underestimated because the answers to the questions depend on the weaver’s ability to recall what happened exactly over the past year. Indeed, the impact of LBP on the reduction of activities and work interruptions was assessed directly by answering the questions in the NQ: (1) Has low back trouble caused you to reduce your activity during the last 12 months? and (2) What is the total length of time (in days) that low back trouble has prevented you from doing your normal work (at home or away from home) during the last 12 months?

Whatever the risk of underestimating the consequences, the results show that it is necessary to implement a strategy to reduce these work interruptions and ensure good living and working conditions.

A correlation analysis was carried out to identify if there are associations between the WRMSDs prevalence and occupational risk factors such as the number of hours worked per day, working experience, and age. The 8-h working day limit imposed by the labour code in Burkina Faso is obviously not respected. Long working hours has already been acknowledged as a source of WRMSDs in the weaving occupation (Durlov et al. 2014; Rahman et al. 2017; Sawadogo et al. 2020). To help weavers avoid working long hours, they should be informed of their negative effects on health and testimonials from people who have suffered from long working hours could, for example, be included in such an approach.

An association between the WRMSD prevalence and working experience is reported (Table 3); similarly other studies show that work-related illness occurs and increases after long exposure to risks (ILO 2013; Rahman et al. 2017; Siddiqui et al. 2021; SPF-EMPLOI 2013). Hence, the interest in taking preventive measures for entry-level workers to anticipate WRMSDs.

The prevalence of low back and knees WRMSDs are associated with the age of the weavers, those who are older than 40 years are more likely to develop WRMSDs (Table 3). Age is a natural phenomenon that reduces physical capacity, muscle strength, endurance, and flexibility, but the demands of the occupation remain the same. This leads to an increased risk of WRMSDs as the demands of the occupation may exceed the physical capabilities of the worker (Chiron et al. 2008; SPF-EMPLOI 2013).

The differences in prevalence within anatomical areas (Table 2) may be explained by a difference in the exposure of these body parts to risk factors in the weaving occupation as the origin of WRMSDs is multifactorial and can come from several sources such as organisation, work techniques, and tools (Dianat & Karimi 2016; Sawadogo et al. 2020). In our study, the assessment of the prevalence of WRMSDs is accompanied by the analysis of the weaving occupation. The results of KIM indicate that the weavers are exposed to several physical and environmental risk factors (Table 4), as shown in the high prevalence of WRMSDs in Table 2. Several risk factors identified here are similar to those of Indian weavers; namely, bent back posture, limited working space, muscular strain, and repetitive limb movements (Nag et al. 2010). Such risk factors have also been identified by other authors who have used biomechanical models to demonstrate associations between low back WRMSDs and biomechanical risk factors, including back bending and twisting, load carrying, muscle fatigue, awkward postures, and frequency of movements (Afshari et al. 2014; Coenen et al. 2013, 2014). These risk factors can lead to potential health consequences such as functional disorders and/or structural damage.

Carrying of heavy loads in the dyeing activity is identified by the KIM as a risk factor (Table 4). These findings are similar to the results of the study conducted by Kadota et al. (2020) on the consequences of heavy load carrying among women in Tanzania (East Africa) showing a substantial burden of MSDs and disability in this population who carry heavy loads daily (Kadota et al. 2020). According to the National Institute for Occupational Safety and Health, there is an acceptable limit of low back compression force and this compression force depends on several factors such as the distance between load and body, the duration of the handling, the frequency of handling, and the weight of the load (NIOSH 1981). Here the weavers use a unimanual technique to carry the buckets filled with yarn and water, so splitting the load could be proposed to decrease the low back compression force (Figure 1).

The risk factor of working in a sitting position should also be considered, as a prolonged sitting position is known to increase pain in cases of LBP (De Carvalho et al. 2020). One can suggest a more comfortable working position or that weavers alternate the sitting position with stretching and/or walking breaks, as the weaving activity is performed while sitting for long hours (Table 1). Finally, the weaving activity is carried out under unfavourable environmental conditions such as heat, glare from daylight, wind, narrow space for movement, and noise (Table 4). Similar results on unfavourable working conditions have been observed in weaving workshops in Asia with an association between the thermal condition of the workshops and low back symptoms (Nazari et al. 2012). Exposure to environmental factors can aggravate, maintain or be a source of WRMSDs for workers (SPF-EMPLOI 2013).

In conclusion, preventive solutions should be implemented in the weaving sector to reduce the high prevalence of WRMSDs (Siddiqui et al. 2021) and limit the occupational risk factors, especially because the resources to manage musculoskeletal conditions are limited in Africa (Ahenkorah et al. 2019; Kaboré, Zanga & Schepens 2022). In addition, particular attention should be given to the informal sector, which is outside the systems that provide prevention, registration and compensation for occupational diseases (ILO 2013).

### Strengths and limitations

Our study highlights the necessity to implement a prevention programme focusing on the reduction of physical stress induced by the workplace layout.

The non-probability sampling method limits the inference of the WRMSDs prevalence on the general population of weavers.

The validity and reliability of the translation of the NQ in Mooré language still needs to be evaluated.

### Implications and recommendations

Our study highlights the issue of work-related musculoskeletal health and the need for prevention in the informal sector, which constitutes a major part of economic activity in low-income countries.

These results can be used to advocate with policymakers to develop specific approaches, including ergonomics, in occupational health and safety programmes and policies in Burkina Faso. Further studies on the beneficial effect of preventive measures are needed.

## Conclusion

Work-related musculoskeletal disorders are common among weavers and LBP is the most frequently cited disorder and the primary reason for work interruptions and reduction of activities. The prevalence of WRMSDs is associated with factors such as the number of hours worked per day, the years of experience and age, but these results need to be confirmed by other studies considering possible confounding factors. Actions based on ergonomic rules are necessary to prevent WRMSDs as the weavers are exposed to several physical and environmental risk factors such as back bending or twisting, load carrying (> 20 kg – 25 kg), awkward shoulder and hand postures, repetitive movements, sitting in forced postures, long working hours, and unfavourable environmental working conditions.

It is hoped that our study will help to implement new strategies to prevent WRMSDs and improve the health status and the quality of life of weavers in Burkina Faso.
